# Supplementary material for: flDPnn: Accurate intrinsic disorder prediction with putative propensities of disorder functions
Source: Nat Commun. 2021 Jul 21;12:4438. doi: 10.1038/s41467-021-24773-7 (PMC8295265; doi:10.1038/s41467-021-24773-7)
Supplement: Supplementary file 1 — Supplementary Information [file 41467_2021_24773_MOESM1_ESM.pdf]

Supplementary Information for fIDPnn: Accurate intrinsic disorder prediction with putative propensities of disorder functions

Gang Hu[1#], Akila Katuwawala[2#], Kui Wang[3#], Zhonghua Wu[3], Sina Ghadermarzi[2], Jianzhao Gao[3] and Lukasz Kurgan[2*]

[1]School of Statistics and Data Science, LPMC and KLMDASR, Nankai University, Tianjin 300071, China
[2]Department of Computer Science, Virginia Commonwealth University, Richmond, VA 23284, USA
[3]School of Mathematical Sciences and LPMC, Nankai University, Tianjin 300071, China
[#] These authors contributed equally
*corresponding author: lkurgan@vcu.edu; +1 804-827-3986

Supplementary Tables

Supplementary Table 1. The *p*-values for Figures 2B and 2E generated on the test dataset. Inspired by CASP, we evaluated statistical significance of differences in predictive performance by resampling half the test dataset 10 times and comparing results for each pair of the five disorder predictors. This assesses whether the improvements offered by the better of the two compared methods are robust to different datasets. We used two-sided paired t-test if the measured values were normal (we assessed normality with the Anderson-Darling test at 0.05 significance); otherwise we applied the Wilcoxon test. Bold font identifies p-values > 0.05.

Prediction target	Methods compared	fIDPnn	ESpritz-D	SPOT-Disorder-Single	IUPred2A-short	IUPred2A-long
**Disordered residues (Figure 2B).** The upper triangle (green background) compares AUC; the lower triangle (blue background) compares F1	**fIDPnn**		1.59E-05	5.34E-05	6.77E-08	2.13E-07
	**ESpritz-D**	2.22E-03		**9.94E-01**	3.62E-06	6.16E-06
	**SPOT- Disorder-Single**	9.66E-03	**9.77E-01**		1.20E-05	2.68E-05
	**IUPred2A-short**	2.14E-06	2.06E-04	1.90E-07		1.00E-02
	**IUPred2A-long**	1.37E-05	1.56E-03	1.95E-06	1.16E-04	
**Fully disordered proteins (Figure 2E).** The upper triangle (green background) compares F1; the lower triangle (blue background) compares MCC	**fIDPnn**		7.63E-03	2.86E-02	2.72E-05	8.83E-04
	**ESpritz-D**	5.03E-03		**4.44E-01**	1.65E-02	**5.75E-01**
	**SPOT- Disorder-Single**	**1.60E-01**	**1.39E-01**		2.91E-04	1.86E-02
	**IUPred2A-short**	5.07E-04	**4.44E-01**	5.63E-03		7.93E-04
	**IUPred2A-long**	1.18E-02	**2.85E-01**	**1.06E-01**	1.83E-02	

Supplementary Table 2. Definition of predictive models for the ablation analysis based on the schematic shown in Figure 1.

Name of model	Protein-level features (sequence-average of the profile values, sequence length, and the distance to each sequence terminus)	Window-level features (profile values for individual residues in window of 5 residues for the disorder prediction and 1 residue for the disorder function prediction)	Residue-level features (average over window of 15 residues for the disorder prediction and 11 residues for the disorder function prediction)	Features computed from disorder function predictions	Features computed from IUPred predictions	Features computed from PSSM generated by PSI-BLAST using the Swiss-Prot dataset
**fIDPnn**	included	included	included	included	included	included
**Residue-level features excluded**	included	included	excluded	excluded for the residue-level features	excluded for the residue-level features	excluded for the residue-level features
**Window-based features excluded**	included	excluded	included	excluded for the window-level features	excluded for the window-level features	excluded for the window-level features
**Protein-level features excluded**	excluded	included	included	excluded for the protein-level features	excluded for the protein-level features	excluded for the protein-level features
**Disorder function predictions excluded**	disorder function predictions excluded	disorder function predictions excluded	disorder function predictions excluded	excluded for the protein-, window- and residue-level features	included	included
**PSSM features excluded**	PSSM features excluded	PSSM features excluded	PSSM features excluded	included	included	excluded for the protein-, window- and residue-level features
**IUPred features excluded**	IUPred features excluded	IUPred features excluded	IUPred features excluded	included	excluded for the protein-, window- and residue-level features	included

Supplementary Table 3. The *p*-values for Figure 3 generated on the test dataset. Inspired by CASP, we evaluated statistical significance of differences in predictive performance by resampling half the test dataset 10 times and comparing results between flDPnn and each of the six ablation variants defined in Suppl. Table S2. This assesses whether the improvements offered by the full version of flDPnn are robust to different datasets. We used two-sided paired t-test if the measured values were normal (we assessed normality with the Anderson-Darling test at 0.05 significance); otherwise we applied the Wilcoxon test. Bold font identifies p-values > 0.05.

**Ablation variants of flDPnn**	**p-values when compared to flDPnn using:**		
	**AUC**	**F1**	**MCC**
Residue-level features excluded	3.82E-06	1.18E-02	1.10E-02
Window-based features excluded	6.11E-06	1.96E-04	7.10E-05
Disorder function predictions excluded	7.42E-03	5.49E-04	8.10E-04
Protein-level features excluded	7.66E-07	7.48E-04	4.11E-04
PSSM features excluded	8.74E-04	1.42E-03	2.20E-03
IUPred features excluded	1.12E-03	**1.37E-01**	**9.41E-02**

Supplementary Table 4. The *p*-values for Figure 4 generated on the test dataset. Inspired by CASP, we evaluated statistical significance of differences in predictive performance by resampling half the test dataset 10 times and comparing results for each pair of the disorder function predictors that target the same function. This assesses whether the improvements offered by the better of the two compared methods are robust to different datasets. We used two-sided paired *t*-test if the measured values were normal (we assessed normality with the Anderson-Darling test at 0.05 significance); otherwise we applied the Wilcoxon test. Bold font identifies p-values > 0.05.

**Prediction target**	**Methods compared**	**flDPnn**	**DisoRDPbind**	**ANCHOR2**	**MorfChibiLight**	**fMoRFpred**
**Disordered protein binding residues** The upper triangle (green background) compares AUC; the lower triangle (blue background) compares F1	**flDPnn**		1.17E-06	2.37E-14	1.57E-04	1.45E-22
	**DisoRDPbind**	6.12E-17		3.59E-10	1.62E-12	5.40E-22
	**ANCHOR2**	7.50E-15	1.65E-14		1.11E-03	1.33E-15
	**MorfChibiLight**	5.39E-18	2.24E-15	5.95E-09		1.57E-15
	**fMoRFpred**	2.24E-25	3.54E-15	1.57E-04	**7.45E-02**	
	**MorfChibi**	1.57E-04	9.21E-07	1.57E-04	**2.04E-01**	1.17E-18

**Prediction target**	**Methods compared**	**flDPnn**	**DisoRDPbind**
**Disordered DNA binding residues** The upper triangle (green background) compares AUC; the lower triangle (blue background) compares F1	**flDPnn**		3.89E-09
	**DisoRDPbind**	**1.25E-01**	

**Prediction target**	**Methods compared**	**flDPnn**	**DisoRDPbind**
**Disordered RNA binding residues** The upper triangle (green background) compares AUC; the lower triangle (blue background) compares F1	**flDPnn**		6.98E-14
	**DisoRDPbind**	**7.83E-02**	

**Prediction target**	**Methods compared**	**flDPnn**	**DFLpred**
**Disordered linker residues** The upper triangle (green background) compares AUC; the lower triangle (blue background) compares F1	**flDPnn**		4.44E-15
	**DFPpred**	2.50E-03	

Supplementary Table 5. Definition of the evaluation metrics.

Short name	Full name	Formula
MCC	Matthews correlation coefficient	$\mathrm{MCC} = \dfrac{TP * TN - FP * FN}{\sqrt{(TP + FP) * (TP + FN) * (TN + FP) * (TN + FN)}}$
F1	F1-measure (harmonic mean of precision and sensitivity)	$\mathrm{F1} = \dfrac{2 * PPR * TPR}{PPR + TPR} = \dfrac{2 * TP}{2 * TP + FP + FN}$
TPR	True positive rate (sensitivity)	$\mathrm{TPR} = \dfrac{TP}{TP + FN}$
FPR	False positive rate	$\mathrm{FPR} = \dfrac{FP}{TN + FP}$
PPR	Predicted positive rate (precision)	$\mathrm{PPR} = \dfrac{TP}{TP + FP}$
TP	True positive	Number of correctly predicted disordered/functional residues
TN	True negative	Number of correctly predicted non-disordered/non-functional residues
FP	False positive	Number of non-disorder/non-functional residues incorrectly predicted as disordered/functional
FN	False negative	Number of disorder/functional residues incorrectly predicted as non-disordered/non-functional

Supplementary Figures

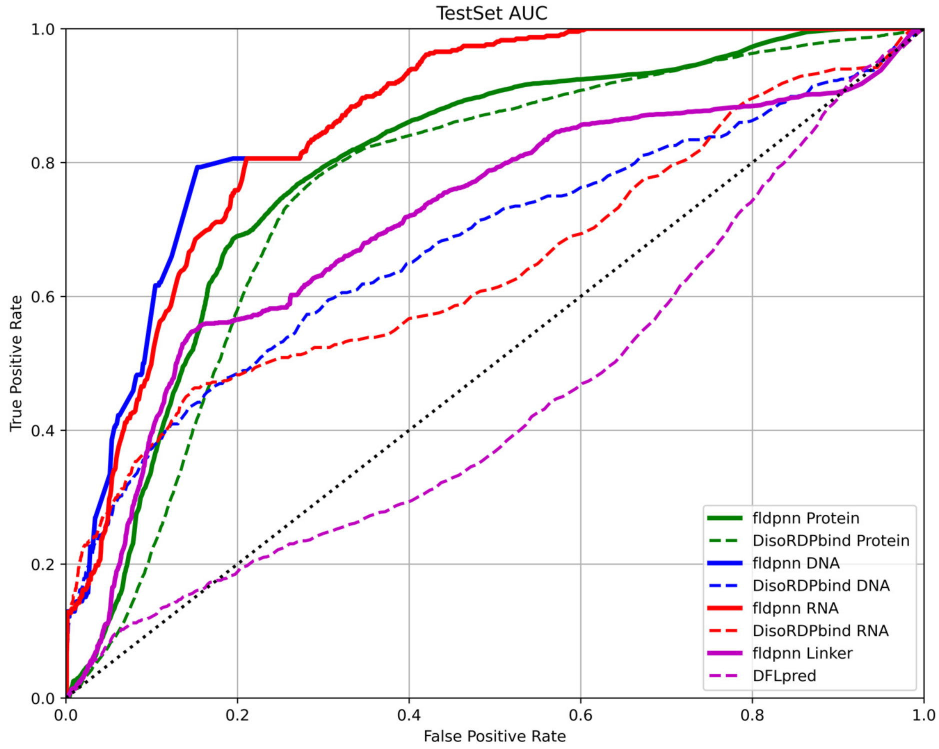

Supplementary Figure 1. **ROC curves and AUC values for the assessment of the quality of the disorder function predictions for the IDRs predicted by flDPnn on the test dataset.** Disorder functions are color-coded. Solid lines represent results for flDPnn. Dashed lines represent results produced by the second-best (according to the AUC scores in Figure 4) function predictor.

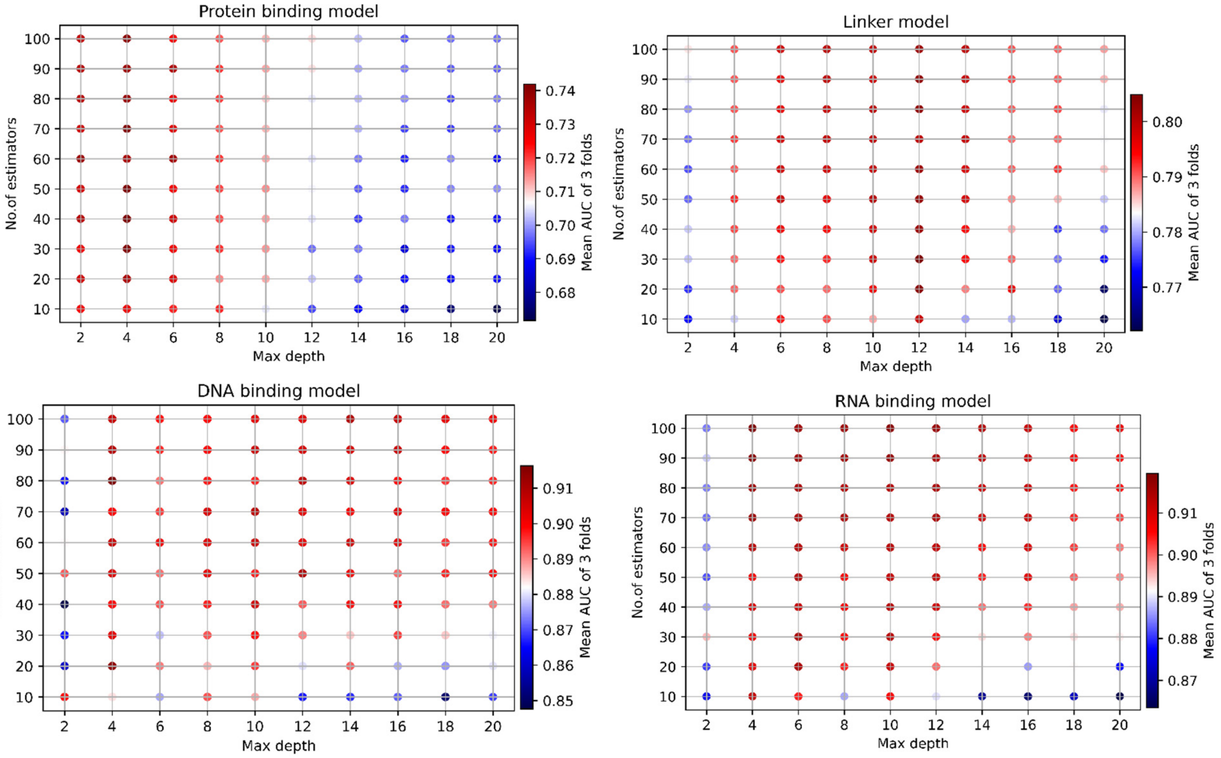

Supplementary Figure 2. Results of the grid search-based parametrization of the random forest models for the prediction of the four disorder functions. We considered two key hyperparameters: number of trees (dubbed number of estimators) = {10,20,30,40,50,60,70,80,90,100} and maximal tree depth = {2,4,6,8,10,12,14,16,18,20}. We implemented the grid search based on the 3-fold cross validation on the training dataset.
