# Supplementary material for: flDPnn: Accurate intrinsic disorder prediction with putative propensities of disorder functions
Source: Nat Commun. 2021 Jul 21;12:4438. doi: 10.1038/s41467-021-24773-7 (PMC8295265; doi:10.1038/s41467-021-24773-7)
Supplement: Supplementary file 3 — Reporting summary [file 41467_2021_24773_MOESM3_ESM.pdf]

nature research

Corresponding author(s): Lukasz Kurgan

Last updated by author(s): Jun 27, 2021

Reporting Summary

Nature Research wishes to improve the reproducibility of the work that we publish. This form provides structure for consistency and transparency in reporting. For further information on Nature Research policies, see our Editorial Policies and the Editorial Policy Checklist.

Statistics

For all statistical analyses, confirm that the following items are present in the figure legend, table legend, main text, or Methods section.

n/a	Confirmed	
☐	☒	The exact sample size (*n*) for each experimental group/condition, given as a discrete number and unit of measurement
☐	☒	A statement on whether measurements were taken from distinct samples or whether the same sample was measured repeatedly
☐	☒	The statistical test(s) used AND whether they are one- or two-sided<br>*Only common tests should be described solely by name; describe more complex techniques in the Methods section.*
☒	☐	A description of all covariates tested
☐	☒	A description of any assumptions or corrections, such as tests of normality and adjustment for multiple comparisons
☒	☐	A full description of the statistical parameters including central tendency (e.g. means) or other basic estimates (e.g. regression coefficient) AND variation (e.g. standard deviation) or associated estimates of uncertainty (e.g. confidence intervals)
☐	☒	For null hypothesis testing, the test statistic (e.g. *F*, *t*, *r*) with confidence intervals, effect sizes, degrees of freedom and *P* value noted<br>*Give P values as exact values whenever suitable.*
☒	☐	For Bayesian analysis, information on the choice of priors and Markov chain Monte Carlo settings
☒	☐	For hierarchical and complex designs, identification of the appropriate level for tests and full reporting of outcomes
☒	☐	Estimates of effect sizes (e.g. Cohen's *d*, Pearson's *r*), indicating how they were calculated

Our web collection on statistics for biologists contains articles on many of the points above.

Software and code

Policy information about availability of computer code

Data collection	Data was collected by using custom-developed scripts written in Python 3 (version 3.8.5).
Data analysis	The training, validation and deployment of the predictive models utilized in flDPnn, which include deep neural net and random forest, were implemented with Python 3 (3.8.5) including the following packages: scikit-learn (0.23.2), keras (2.4.3), tensorflow (2.4.1) and pandas (1.2.2). We also used plotly (4.14.3) to visualize results on the webserver, PSIPRED (4.02) (http://bioinfadmin.cs.ucl.ac.uk/downloads/psipred/) for the secondary structure prediction, BLAST (2.2.24) (https://blast.ncbi.nlm.nih.gov/Blast.cgi?CMD=Web&PAGE_TYPE=BlastDocs&DOC_TYPE=Download) to produce PSSM, IUPred (1.0) (https://iupred2a.elte.hu/download_new) to predict disorder, DisoRDPbind (1.0) (http://biomine.cs.vcu.edu/servers/DisoRDPbind/) to predict disordered binding residues, DFLpred (1.0) (http://biomine.cs.vcu.edu/servers/DFLpred/) to predict linker regions, and fMoRFpred (1.0) (http://biomine.cs.vcu.edu/servers/fMoRFpred/) to predict MoRF regions.<br>The complete software package is available at https://gitlab.com/sina.ghadermarzi/fldpnn. We also include docker container that simplifies local installation at https://gitlab.com/sina.ghadermarzi/fldpnn_docker.

For manuscripts utilizing custom algorithms or software that are central to the research but not yet described in published literature, software must be made available to editors and reviewers. We strongly encourage code deposition in a community repository (e.g. GitHub). See the Nature Research guidelines for submitting code & software for further information.

Data

Policy information about availability of data

All manuscripts must include a data availability statement. This statement should provide the following information, where applicable:

- Accession codes, unique identifiers, or web links for publicly available datasets
- A list of figures that have associated raw data
- A description of any restrictions on data availability

Data was collected by parsing the publicly available DisProt repository (https://www.disprot.org/). This includes the training, validation, test and CAID datasets. The parsed datasets (including raw data and identifiers) are freely available (no restrictions) at http://biomine.cs.vcu.edu/servers/flDPnn/. We also utilize the publicly available SwissProt dataset (https://www.uniprot.org/statistics/Swiss-Prot) to produce PSSM.

Field-specific reporting

Please select the one below that is the best fit for your research. If you are not sure, read the appropriate sections before making your selection.

- [x] Life sciences
- [ ] Behavioural & social sciences
- [ ] Ecological, evolutionary & environmental sciences

For a reference copy of the document with all sections, see nature.com/documents/nr-reporting-summary-flat.pdf

Life sciences study design

All studies must disclose on these points even when the disclosure is negative.

Sample size	Training dataset: 445 proteins. Validation dataset: 100 proteins. Test dataset: 200 proteins. Benchmark CAID dataset: 646 proteins. Sample size calculations are not performed when training machine learning models that make amino acid-level predictions since datasets are inherently very big. We follow the best practice in the field where the datasets include at least 100 proteins, which accounts for at least 20,000 samples (i.e., predictions are trained and made for each amino acid in a given protein sequence).
Data exclusions	There was not data exclusions since the data was collected from manually curated sources: DisProt and Swiss-Prot.
Replication	This work covers the development and validation of computational methods, with no experimental biological data involved. Results produced by computational models are deterministic (repeated run produce the same results), and thus replication was not necessary.
Randomization	Samples (proteins) were assigned at random into the corresponding datasets. There is no need to accommodate for covariates.
Blinding	This work covers the development and validation of computational methods and thus blinding is not applicable.

Reporting for specific materials, systems and methods

We require information from authors about some types of materials, experimental systems and methods used in many studies. Here, indicate whether each material, system or method listed is relevant to your study. If you are not sure if a list item applies to your research, read the appropriate section before selecting a response.

Materials & experimental systems

n/a	Involved in the study
x	Antibodies
x	Eukaryotic cell lines
x	Palaeontology and archaeology
x	Animals and other organisms
x	Human research participants
x	Clinical data
x	Dual use research of concern

Methods

n/a	Involved in the study
x	ChIP-seq
x	Flow cytometry
x	MRI-based neuroimaging
